# Primary pulmonary mucoepidermoid carcinoma: an analysis of 21 cases

**DOI:** 10.1186/1477-7819-10-232

**Published:** 2012-11-01

**Authors:** Jun-jie Xi, Wei Jiang, Shao-hua Lu, Chun-yan Zhang, Hong Fan, Qun Wang

**Affiliations:** 1 Division of Thoracic Surgery, Zhongshan Hospital, Fudan University, 180 Fenglin Road, Shanghai, 200032, China; 2 Division of Pathology, Zhongshan Hospital, Fudan University, 180 Fenglin Road, Shanghai, 200032, China; 3 Division of Laboratory Medicine, Zhongshan Hospital, Fudan University, 180 Fenglin Road, Shanghai, 200032, China

**Keywords:** Pulmonary mucoepidermoid carcinoma, Treatment, Prognosis

## Abstract

**Background:**

The optimal treatment for pulmonary mucoepidermoid carcinoma (MEC), a rare type of tumor, has not been established yet. This study analyzed the survival of pulmonary MEC patients and attempted to find clues for optimal treatment.

**Methods:**

A total of 21 patients with pulmonary MEC from November 2004 to January 2011 were included in the investigation. Immunohistochemistry, epidermal growth factor receptor (EGFR) mutation, and survival were retrospectively studied.

**Results:**

Among the 21 pulmonary MEC patients, 17 were diagnosed with low-grade malignancy and 4 with high-grade malignancy through pathological examination. The prognosis was found to be poor in the presence of lymph nodes. The expression rates of EGFR and HER2 were 28.6% and 0%, respectively, which correlated with neither grade nor prognosis. The mutation rate of EGFR was 0. Log-rank test results indicated that age, grade, lymph node metastasis, and tumor-node-metastasis stage were prognostic factors.

**Conclusion:**

Age, grade, lymph node metastasis and tumor-node-metastasis stage correlate with the survival of pulmonary MEC patients.

**Trial registration:**

This study was approved and registered by the Ethics Committee of Zhongshan Hospital. Written informed consent was obtained from all participants prior to treatment.

## Background

Pulmonary mucoepidermoid carcinoma (MEC) is rare, accounting for approximately 0.2% of all malignant lung tumors [1]. This tumor is believed to be indolent; however, little is known about its clinical features because of the low incidence rate. No consensus on optimal treatment strategy is available, and surgery is the common choice [2]. Treatment involving epidermal growth factor receptor (EGFR) tyrosine kinase inhibitors (TKIs) showed promising outcome for MEC patients [3]. However, these results need confirmation. In the present study, we detected the expression rates of EGFR and HER2 of MEC, analyzed EGFR mutation status, and explored the prognostic factors of survival. The results of the present study may serve as a basis for the development of a treatment strategy for MEC.

## Methods

### Clinical features, immunohistochemistry, and EGFR mutation analysis

Patients who presented with pathologically confirmed primary pulmonary MEC at the Division of Thoracic Surgery, Zhongshan Hospital between November 2004 and January 2011 were enrolled in this study. This study was approved by the Ethics Committee of Zhongshan Hospital. Written informed consent was obtained from all participants prior to treatment. Follow-ups were conducted via telephone interview by contributing physicians. For additional analyses, the data were analyzed anonymously, wherein informed consent was not required. Table 1 describes the clinical features of the 21 patients.

**Table 1:** Clinical features of patients

	**Number**
Male:female	10:11
Age (mean)	43.4
Smoking	2/21
Initial symptoms	
Cough	7
Bloody sputum	3
Hemoptysis	2
Cough and bloody sputum	2
Cough and hemoptysis	1
Dyspnea	1
Chest pain	1
Asymptomatic	4
Tumor location	
Right main bronchus	1
Left main bronchus	2
Intermediate bronchus	2
Right upper lobe	3
Right middle lobe	2
Right inferior lobe	5
Left upper lobe	4
Left inferior lobe	2

Treatment and survival were retrospectively analyzed. Paraffin-embedded tissues of tumors were stained for the detection of EGFR and HER2 expressions by immunohistochemistry. Novocastra liquid mouse monoclonal antibody epidermal growth factor receptor (Leica Biosystems, Newcastle, UK) and polyclonal rabbit anti-human c-erbB-2 oncoprotein (Dako, Glostrup, Denmark) for EGFR and HER-2, respectively, were provided by the Division of Pathology of our hospital. EGFR and HER2 expressions were evaluated according to previously described criteria [4,5].

Exons 18-21 of EGFR were examined by direct sequencing. The analyzed sites were G719S, exon 19, T790M, L858R, and L861Q.

### Statistical analysis

Cumulative survival rates were calculated by the Kaplan-Meier method, and the survival curves were compared using the log-rank test. The chi-square test was performed to determine the relationship among EGFR/HER2 expression, differentiation grade, and lymph node metastasis. Univariate and multivariate analyses using Cox's proportional hazard model were conducted to obtain the risk factors of MEC-related death and recurrence. The multivariate analysis was conducted by forward stepwise regression. All analyses were performed using SPSS software version 13.0 (SPSS Inc., Chicago, IL, USA).

## Results

### Treatments and pathology

None of the patients was given neoadjuvant therapy, and all patients underwent surgery. Lobectomies were performed on 13 patients. The other patients underwent bilobectomy (n = 2), sleeve lobectomy (n = 2), segmental lung resection (n = 1), local resection (n = 1), bronchial sleeve resection and reconstruction (n = 1), and pneumonectomy with resection and reconstruction of superior vena cava and atrium (n = 1).

**Figure 1:**
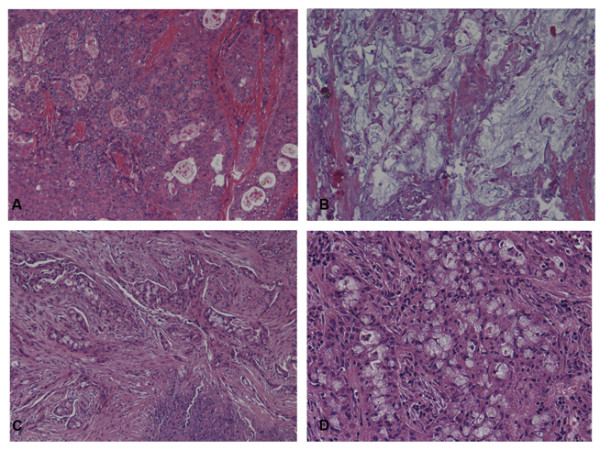
**Representative images of hematoxylin and eosin-stained pathologic specimens.** Low-grade tumor was composed of epideroid, intermediate, columnar, and goblet cells. The goblet cells were rich in mucin. (**A**) Low-grade mucoepidermoid carcinoma (MEC), 100×; (**B**) low-grade MEC, 400×. The cellular component of high-grade tumor was the same as that of low-grade tumor, but squamoid cells were predominant. Cytologic atypia and mitotic activity were more common in high-grade tumor. (**C**) High-grade MEC, 100×; (**D**) high-grade MEC, 400×.

Postoperative pathology showed low-grade MEC in 17 patients and high-grade MEC in 4 patients (Figure 1) according to the criteria described by Yousem and Hochholzer [6]. Based on the tumor-node-metastasis (TNM) staging system of the American Joint Committee on Cancer and the International Union against Cancer (7th edition, 2009), more than half of the patients were in stage I (Table 2). Lymph node dissection was not performed in two cases.

**Table 2:** Pathology and survival information

**Number**	**Grade**	**Stage**	**T**	**N**	**M**	**EGFR IHC**	**EGFR mutation**	**Recurrence metastasis**	**Survival status**	**OS/month/month**	**PFS/month/month**
1	Low	Ia	1a	0	0	50%++	N/A	-	Alive	29	29
2	Low	Ia	1a	0	0	0	-	-	Alive	76	76
3	Low	Ia	1a	0	0	0	-	-	Alive	77	77
4	Low	Ia	1b	0	0	0	-	-	Alive	62	62
5	Low	Ia	1b	0	0	0	-	-	Alive	5	5
6	Low	Ib	2a	0	0	30%+	-	-	Alive	22	22
7	Low	Ib	2a	0	0	0	-	-	Alive	46	46
8	Low	Ib	2a	0	0	0	-	Recurrence	Dead	41	40
9	Low	Ib	2a	0	0	90%+	-	-	Alive	66	66
10	Low	Ib	2a	0	0	0	-	-	Alive	67	67
11	Low	Ib	2a	0	0	0	-	-	Alive	67	67
12	Low	IIa	2a	1	0	20%+	-	-	Alive	29	29
13	Low	IIb	3	0	0	0	-	-	Alive	5	5
14	Low	IIb	3	0	0	0	-	-	Alive	54	54
15	Low	IIIb	4	2	0	90%++	-	-	Perioperative dead	-	-
16	Low	-	3	x	0	0	N/A	-	Alive	74	74
17	Low	-	1b	x	0	0	-	-	Alive	25	25
18	High	Ia	1a	0	0	0	-	-	Alive	47	47
19	High	IIb	2b	1	0	100%+	-	Contralateral lung metastasis	Dead	65	63
20	High	IIIa	2a	2	0	0	-	Recurrence	Dead	31	30
21	High	IIIa	2a	2	0	0	-	Liver metastasis	Dead	23	15

*EGFR* epidermal growth factor receptor, *IHC* immunohistochemistry, *N/A* not available, *OS* overall survival, *PFS* Progression-free survival, *TNM* tumor-node-metastasis.

One patient (Number 14) received adjuvant radiotherapy because the surgical margin was positive. One patient (Number 12) with tumor (T2aN1M0) received adjuvant chemotherapy because of the physician’s advice.

### Expressions of EGFR and HER2

**Figure 2:**
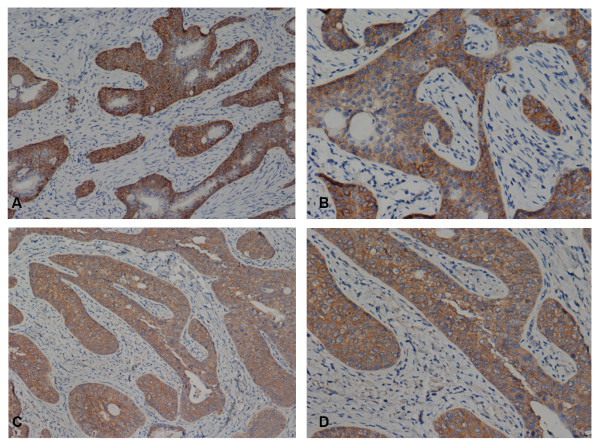
**Representative images of immunohistochemistry staining.** (**A**) Positive EGFR expression, 100×; (**B**) positive EGFR expression, 400×; (**C**) false-positive HER2 expression because only cytoplasmic staining was observed, 100×; (**D**) false-positive HER2 expression because only cytoplasmic staining was observed, 400×.

Table 2 describes the expression of EGFR, and the specimen was considered positive if the percentage/intensity was >10%+. The positive rates of EGFR and HER2 were 28.6% (6/21) and 0%, respectively (Figure 2). The expression of EGFR was not associated with differentiation (*P* = 1) or lymph node metastasis (*P* = 0.28).

### EGFR mutation

Among the 21 pulmonary MEC cases, 19 were available for EGFR mutational analysis. All the tumors examined were negative for EGFR mutation.

### Survival

One patient (Number 15) died in the hospital because of tumor invasion at the carina, right pulmonary artery, superior vena cava, and right atrium. The tumor was low grade and was at stage IIIB. As mentioned previously, the patient received pneumonectomy with resection and reconstruction of the superior vena cava and atrium. At 24 days after surgery, the patient died of respiratory failure. Follow-up information for the other 20 patients is listed in Table 2.

The follow-up period for the 16 low-grade tumor cases ranged from 5 to 77 months (mean: 46.6 months). The average age of the patients was 38.8 years. Among these patients, 15 survived and 1 died. The patient (Number 8) was at stage Ib (T2N0M0). His disease recurred at 40 months after surgery, no subsequent treatment was given, and he died a month later.

The follow-up period for the four high-grade tumor cases ranged from 23 to 65 months (mean: 41.5 months). The average age of the patients was 62.0 years. Among these patients, three died and one survived. Patient Number 19 (stage IIb, T2bN1M0) had contralateral lung metastasis at 63 months after surgery and then died 2 months later. Patient Number 20 (stage IIIa, T2aN2M0) experienced a recurrence at 30 months after surgery and died a month later. Both patients mentioned earlier did not receive any subsequent treatment. Patient Number 21 was at stage IIIa (T2aN2M0). After disease metastasized to the liver at 15 months after surgery, the patient started with gefitinib treatment (250 mg/d). The patient died eight months later.

**Figure 3:**
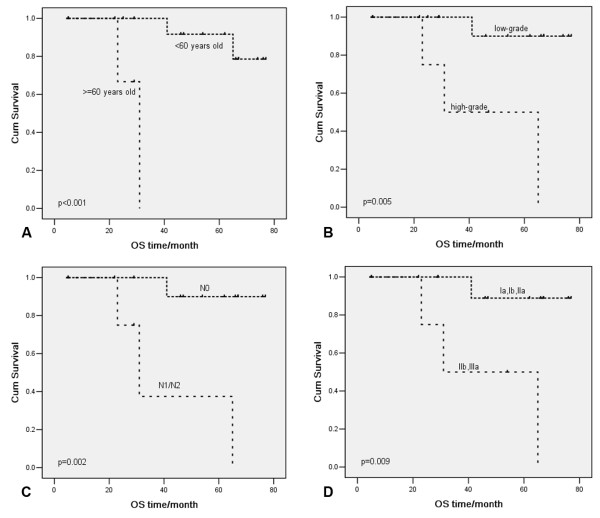
**Kaplan-Meier curves of overall survival (OS) for variates: (A) age, (B) grade, (C) lymph node metastasis, and (D) TNM stage**.

**Figure 4:**
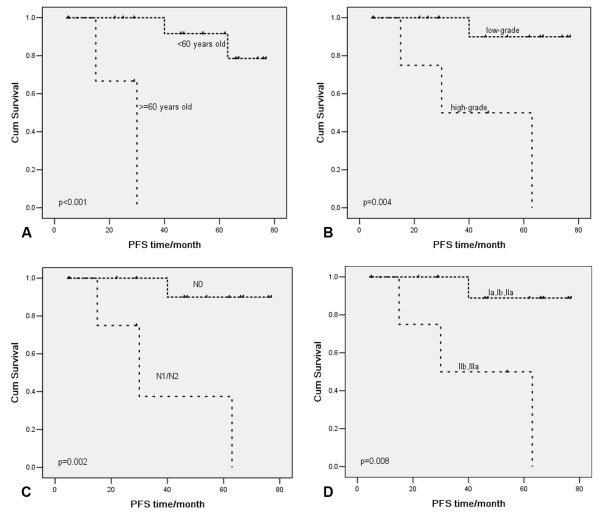
**Kaplan-Meier curves of progression-free survival (PFS) for variates:** (**A**) age, (**B**) grade, (**C**) lymph node metastasis, and (**D**) TNM stage.

Kaplan-Meier curve of overall survival (OS) (Figure 3) and progression-free survival (PFS) (Figure 4) showed that age, differentiation grade, lymph node metastasis, and TNM stage were associated with OS and PFS. Univariate Cox proportional hazard regression analyses showed that age, grade, lymph node metastasis, and TNM stage were prognostic factors (Table 3). Age, grade, lymph node metastasis, and TNM stage were included in the multivariate analysis. Lymph node metastasis was the only variate in the regression equation of OS (hazard ratio (HR) = 0.06, 95% confidence interval (95% CI): 0.01 to 0.62, *P* = 0.018) and PFS (HR = 0.06, 95% CI: 0.01 to 0.61, *P* = 0.017).

**Table 3:** Hazard ratio of variates for overall survival and progression-free survival

	**Overall survival**			**Progression-free survival**		
	**HR**	**95% CI**	***P *value**	**HR**	**95% CI**	***P *value**
Age	1.22	1.01-1.48	0.044	1.22	1.01-1.48	0.044
EGFR	0.80	0.08-7.82	0.845	0.85	0.09-8.38	0.888
Grade	0.08	0.01-0.75	0.028	0.08	0.01-0.74	0.026
T	0.93	0.10-8.98	0.946	0.91	0.10-8.88	0.937
N	0.06	0.01-0.62	0.018	0.06	0.01-0.61	0.017
TNM stage	0.09	0.01-0.87	0.037	0.09	0.01-0.85	0.036

*CI* confidence interval, *EGFR* epidermal growth factor receptor, *HR* hazard ratio, *TNM* tumor-node-metastasis.

## Discussion

Pulmonary MEC, which was firstly reported by Smetana *et al*. [7], was derived from the exocrine duct of the tracheobronchial submucosal glands. The clinical features of the 21 patients were similar to the results reported previously [6,8]. Making an exact diagnosis of pulmonary MEC before surgery is difficult because the clinical manifestations and auxiliary examination findings are unspecific. High-grade MEC is often difficult to distinguish from adenosquamous carcinoma. Keratinization, a feature of adenosquamous carcinoma, is absent in high-grade MEC. Another difference is that the surface epithelium of MEC rarely shows *in situ* carcinoma. We performed immunohistochemistry staining to ensure the diagnosis. TTF-1 and surfactant are commonly positive in adenosquamous carcinoma, while they are always negative in high-grade mucoepidermoid carcinoma of lung.

The tumor is classified as either low grade or high grade according to histology appearance, cellular atypia, mitotic activity, local invasion, and necrosis. The biological behavior of pulmonary MEC was believed to be associated with differentiation [6]. Well-differentiated tumors present benign behavior, and vice versa. The prognosis of low-grade tumor patients who underwent surgery is better than that of high-grade tumor patients who underwent surgery. However, none of the previous studies mentioned HR. Comparing the prognosis of poorly differentiated and well/moderately differentiated non-small cell lung cancer (NSCLC), HR was 1.15 (95% CI, 0.81 to 1.64, *P* = 0.42), which is not statistically significant [9]. In this research, according to the Kaplan-Meier curve and HR, low-grade tumor cases had advantage over high-grade tumor cases in OS and PFS.

Yousem and Hochholzer [6] proposed that the prognosis of older patients is worse and that hilar lymph node metastasis indicates worse prognosis. In the series reported by Vadasz and Egervary [2], only high-grade tumors develop lymph node metastasis. In the present study, univariate analysis showed that age and TNM stage were correlated with OS and PFS. However, multivariate regression analysis revealed that lymph node metastasis was the only independent prognostic factor. It should be noticed that multivariate analysis should be based on a large sample, and our study is limited by the small sample. We are just trying to get clues for treatment and prognosis, and we can only assume that maybe lymph node metastasis is the most important prognostic factor of pulmonary MEC.

To date, surgery is the preferred treatment of pulmonary MEC, in which tumor location determines the surgical procedure. Among the 21 cases, 16 tumors were found in the lobar bronchi, for which lobectomy was the most common surgical procedure, accounting for 61.9% (13/21) of the cases. Sleeve lobectomy should be considered when the primary bronchus is invaded, and pneumonectomy is the last choice. In this investigation, only one patient underwent pneumonectomy because of tumor invasion at the carina, right pulmonary artery, superior vena cava, and right atrium.

No evidence proved the effect of chemotherapy or radiotherapy. Some studies reported that chemotherapy or radiotherapy is inefficient [1,10]. Although paclitaxel is active in MEC of salivary glands [11], its effectiveness in pulmonary MEC has yet to be determined. Therefore, some clinicians [2] do not propose adjuvant chemotherapy or radiotherapy. Our present data showed that 3/4 of high-grade pulmonary MEC patients developed lymph node metastasis, and the prognosis was dismal. As a type of NSCLC, adjuvant therapy was indicated for pulmonary MEC when lymph node metastasis occurred. Thus, the treatment of pulmonary MEC with lymph node metastasis using surgery alone poses some problems. Although the prognosis of patients with lymph node involving pulmonary MEC was poor, no subsequent therapy was proven to be optimal. We supposed that treatment involving EGFR TKIs may be a choice for pulmonary MEC. In a previous study, a pulmonary MEC patient received gefitinib after the tumor metastasized to chest wall and contralateral lung [3]. CT scans revealed that the metastatic lesions responded to gefitinib and that no EGFR tyrosine kinase mutation was detected in the chest wall mass. In the present study, patient Number 21 had a prolonged survival period of eight months after gefitinib administration. Patient Numbers 19 and 20 survived for two months and one month after progression without any subsequent treatment, respectively.

Assuming that EGFR/HER2 was associated with survival, we analyzed the expressions of EGFR and HER2 in the 21 cases. The lack of HER2 expression was coincident with the previous results. EGFR expression was not common either in the lung or salivary glands, which differed from the results of previous studies [12,13]. EGFR expression was correlated with neither differentiation grade nor prognosis. In MEC of salivary glands, EGFR expression was common, whereas HER-2 expression was not [12]. Han *et al*. [3] detected EGFR and HER-2 expressions in six pulmonary MEC patients, with positive rates of 4/6 and 0, respectively. Macarenco *et al*. [13] reported that 92% (11/12) of pulmonary MEC specimens were positive for EGFR expression. In MEC of salivary glands, EGFR expression is correlated with histological grade but not with patient outcome [14].

The relationship between EGFR expression and prognosis in NSCLC is disputable, and EGFR mutation status is considered as a strong predictor for EGFR TKI administration [15]. A previous study reported that MEC has EGFR mutation in 40% (2/5) of cases [3]. By contrast, EGFR mutation was negative in the present research. Although the results were negative, we cannot conclude that treatment involving EGFR TKIs is ineffective for MEC. NCI-H292 cell line, a pulmonary MEC cell line with wild-type EGFR, demonstrates enhanced sensitivity to gefitinib compared with other NSCLC lines with wild-type EGFR [16]. EGFR is not the only target of gefitinib. Some clinicians [17] assumed that t(11;19) and associated fusion CRTC1-MAML2 may be the targets of EGFR TKI therapy. However, further studies need to be conducted to clarify the mechanism of EGFR TKIs.

## Conclusion

Age, grade, lymph node metastasis and tumor-node-metastasis stage correlate with the survival of pulmonary MEC patients.

## Competing interests

The authors declare that they have no competing interests.

## Authors’ contributions

Xi JJ collected information of the cases, carried out statistic analysis, conducted follow-ups, and drafted the manuscript. Jiang W collected information of the cases and participated in the revision of the manuscript. Lu SH verified the diagnosis and performed immunohistochemistry. Zhang CY carried out the detection of gene mutation. Fan H participated in collecting information of cases and manuscript drafting. Wang Q conceived the article and participated in the manuscript drafting. All authors read and approved the final manuscript.
